# The Value of Brain Structural Magnetic Resonance Imaging Combined with APOE--*ε*4 Genotype in Early Diagnosis and Disease Progression of Senile Vascular Cognitive Impairment No Dementia

**DOI:** 10.1155/2022/8613024

**Published:** 2022-03-05

**Authors:** Weina Zhao, Changhao Yin, Fengbo Yu, Lifei Wan, Siyao Che, Lili Cui

**Affiliations:** ^1^Department of Neurology, The People's Hospital of Gaozhou, Guangdong Medical University, Maoming 525200, China; ^2^Department of Neurology, Hongqi Hospital Affiliated to Mudanjiang Medical University, Heilongjiang,157011, China; ^3^Heilongjiang Key Laboratory for Prevention and Treatment of Ischemic Stroke, Heilongjiang,157011, China; ^4^Drug Clinical Trial Institution, The People's Hospital of Gaozhou, Guangdong Medical University, Maoming 525200, China; ^5^Department of General Surgery, The People's Hospital of Gaozhou, Guangdong Medical University, Maoming 525200, China; ^6^Department of Neurology, Laboratory of Age-Related Cardiac and Cerebral Diseases, Affiliated Hospital of Guangdong Medical University, Zhanjiang, China

## Abstract

**Objective:**

To explore the value of brain structure magnetic resonance imaging combined with APOE-*ε*4 genotype in the early diagnosis and disease progression of elderly patients with vascular cognitive impairment no dementia (VCIND).

**Methods:**

The first stroke patients admitted to our hospital from March 2017 to December 2018 were collected, including 130 cases of vascular cognitive impairment no dementia (VCIND group) and 50 cases of the control group (NC group). The basic information of all subjects was recorded, and APOE-*ε*4 alleles of all subjects were detected. The neuropsychological test scale evaluated the cognitive psychology of the subjects, and they were scanned by multi-parameter MRI. After follow-up, VCIND patients were divided into the dementia group and the nondementia group. MRI scans were again performed, and the risk factors of VCIND patients developing dementia were analyzed.

**Results:**

Compared with the NC group, patients in the VCIND group had shorter years of education, more patients with hypertension, higher levels of homocysteine (Hcy), and lower cognitive ability. Patients with White Matter Volume (WMV), White Matter Hyperintensity (WMH), Lacunar Infarction (LI), elevated Fazekas scores, and APOE-*ε*4 gene carriers are more likely to develop VCIND. After 12 months of follow-up, compared with the nondementia group, the number of WMV, WMH, Fazekas scores, and APOE-*ε*4 gene carriers in the dementia group was significantly increased. In addition, the progression-free survival rate of APOE-*ε*4 gene carriers was significantly lower than that of nonAPOE-*ε*4 gene carriers.

**Conclusion:**

Years of education, hypertension, high levels of Hcy, elevated WMV, WMH, LI, and Fazekas scores, and carrying the APOE-*ε*4 gene are risk factors for VCIND in stroke patients. Craniocerebral structural MRI combined with APOE-*ε*4 genotype has a diagnostic role in the early diagnosis and disease progression of elderly patients with VCIND.

## 1. Introduction

Vascular cognitive impairment (VCI) is a syndrome ranging from mild cognitive impairment to dementia and is the second cause of Alzheimer's disease in China [1]. It is reversible in the early stage and is currently one of the preventable dementia diseases. According to different diagnostic criteria, VCI mainly includes nondementia VCI (VCIND), vascular dementia (VD), and mixed dementia (MD) [2]. VCIND is an early stage of mild cognitive impairment caused by cerebrovascular injury. Its condition is hidden, and its degree of damage to cognitive function has not reached the criteria of dementia. The most common damage sites are the cerebral subcortex and frontal lobe, but as the disease progresses, many VCINDs can develop into VD [3]. At present, the therapeutic effect of drug intervention for VD is still not ideal. Therefore, it is of great significance to actively carry out research and early prevention and treatment of VCIND, improve early symptoms, and delay the progression of the disease course for the prevention and treatment of VD.

At present, the diagnosis of VCIND is still based on clinical manifestations and neuropsychological scales, which are subjective and are not conducive to early clinical diagnosis and prevention. With the in-depth study of multiple magnetic resonance imaging (MRI) sequences, brain CT and MRI can also better present lacunar cerebral infarction in cerebral small vessel disease. This technology has now received extensive attention [4]. VCIND is mainly caused by leukoaraiosis, but routine examinations such as CT and MRI routine sequence (T1T2, DWI, and FLAIR sequence) can only provide a small amount of information on the white matter conduction tract, and the clinical diagnostic value is limited. Therefore, finding new diagnostic methods is of great significance for the treatment of VCIND.

The apolipoprotein *E* (ApoE) gene is located on human chromosome 19q13.2, which plays an important role in maintaining and repairing nerve cells. Its gene is located on chromosome 19 and is encoded by three alleles, namely ApoE-*ε*2, ApoE-*ε*3, and ApoE-*ε*4, of which ApoE-*ε*4 is the largest genetic risk factor for Alzheimer's disease (AD) [5–7]. More than half of AD patients carry ApoE-*ε*4, and ApoE-*ε*4 alleles can accelerate the occurrence of cognitive dysfunction [8]. Studies have shown that the mechanisms by which ApoE-*ε*4 increases the risk of AD are multifaceted, including amyloid *β* (A*β*)-dependent effects, that is, regulating A*β* levels, neurotoxicity and neuroinflammation, the development of neurons that are not related to A*β*-dependent effects, glucose metabolism, brain activity, and lipid metabolism [9]. Studies have reported that ApoE-*ε*4 may increase the risk of VCIND in Chinese Han ethnic female patients [10]. Han et al. also confirmed that the ApoE-*ε*4 allele is a risk factor related to VCIND after cerebral infarction [11]. However, there are few studies on the influence of the ApoE-*ε*4 allele on the progression of VCIND. Therefore, this study will combine brain structural MRI and the detection of ApoE-*ε*4 alleles to explore the value of these two in the early diagnosis and disease progression of VCIND elderly patients.

## 2. Materials and Methods

### 2.1. Research Subjects

The first stroke patients admitted to our hospital from March 2017 to December 2018 were collected. According to whether cognitive dysfunction occurred, all patients were divided into the VCIND group (*n* = 130) and the control group (NC group, *n* = 50). The Hongqi Hospital Affiliated to Mudanjiang Medical University committee approved this study (no. 202114). Patients and their families understood the research and signed the informed consent form.

Inclusion criteria: (1) all subjects aged 65–80 years old; (2) the interval between stroke onset and hospital visit is 3 to 12 months; (3) the duration of cognitive impairment is 3 months or more; (4) Clinical Dementia Rating (CDR) ≥ 0.5 and Activities of Daily Living Scale (ADL) score <20; (5) the patient's cognitive impairment or memory impairment is caused by cerebrovascular disease; (6) no mental disorders, such as anxiety and depression within 1 month of the examination; [7] all subjects voluntarily participated in this study, and they all knew and agreed with the circumstances.

Exclusion criteria: (1) cognitive dysfunction caused by other factors, such as Alzheimer's disease and Parkinson's disease; (2) patients with mental illness, intracranial tumors, and epilepsy; (3) physical disability, severe hearing, speaking, and visual impairment individuals; (4) a history of serious physical disease; (5) severe claustrophobia and MRI contraindications (such as pacemakers and metal foreign bodies); (6) patients who do not cooperate with the examination.

### 2.2. Information Collection

The basic information of all subjects was collected as follows: age, gender, body mass index (BMI), years of education, stroke onset time, hypertension, diabetes, fasting blood glucose, coronary heart disease, smoking history, drinking history, triglyceride (TG), cholesterol (TC), high-density lipoprotein (HDL), low-density lipoprotein (LDL), and homocysteine (Hcy) levels.

### 2.3. APOE-*ε*4 Allele Typing

Five milliliters of anticoagulated venous blood were taken, and DNA was extracted according to the genomic DNA extraction kit (QIAGEN) instructions. PCR amplified the target DNA according to the instructions of the PCR amplification kit (Promega), and the reaction volume was 20 *μ*l. The amplified products were digested using the HhaI digestion kit (Thermo) with a reaction volume of 30 *μ*l. The digested product was stained after polyacrylamide gel electrophoresis and compared with the standard molecular weight. The ApoE genotype was determined according to the number of bands. The primer sequence of APOE-*ε*4 is listed in Table 1.

### 2.4. Cognitive Psychological Assessment

Neuropsychological test scales were used to test the cognitive psychology of all subjects, mainly including Clinical Dementia Rating (CDR) Scale [12], Activities of Daily Living Scale (ADL) [12], Montreal Cognitive Assessment (MoCA) Scale [13], Hamilton Rating Scale for Depression (HAMD) [14], and Elderly Cognitive Abilities Screening Instrument (CASI) [15].

### 2.5. Multi-Parameter MRI Imaging (MP-MRI)

Brain MRI scan was performed using a magnetic resonance imaging scanner, including transverse and sagittal T1WI, transverse and coronal T2WI, and transverse FLAIR. White matter volume (WMV), white matter hyperintensity (WMH), lacunar infarction (LI), areas of leukoaraiosis (frontal region, parieto-occipital region, temporal region, and basal ganglia region), degree of white matter damage, and cerebral atrophy were recorded. The Fazekas score assessed the degree of white matter damage [16].

### 2.6. Follow-Up

After diagnosis and treatment in our hospital, patients in the VCIND group were followed up for 12 months. According to whether the patient developed dementia, the patients were divided into the dementia group (*n* = 29) and the nondementia group (*n* = 101). Cognitive psychological assessments were performed on VCIND patients at 3 months, 6 months, and 12 months after discharge, and patients who progressed to dementia were recorded. The number of increases in patients with dementia was calculated. On the 12th month after discharge, the patient was examined again by MP-MRI.

### 2.7. Statistical Analysis

The data were statistically analyzed using SPSS 22.0 software. The measurement data satisfied the normal distribution and were represented by the mean ± standard deviation (mean ± SD). The independent sample *t*-test was used for comparison between groups. When the data did not meet the normal distribution, it was represented by Median (P25, P75), and the Mann–Whitney *U* test was used for comparison between groups. The enumeration data were expressed as *n* (%), and the chi-square test was used for comparison between groups. Binary logistic regression was used to analyze the risk factors of VCIND development. Kaplan–Meier was used to calculate the survival rate and draw the survival curve. *P* < 0.05 indicated a statistically significant difference.

## 3. Results

### 3.1. General Patient Information

First, we statistically analyzed the baseline data of patients. As listed in Table 2, there was no significant difference in the age, gender, BMI, stroke onset time, diabetes, fasting blood glucose, coronary heart disease, smoking history, drinking history, triglyceride (TG), cholesterol (TC), high-density lipoprotein (HDL), and low-density lipoprotein (LDL) levels between the NC group and the VCIND group (*P* > 0.05). However, compared with the NC group, patients in the VCIND group had fewer years of education, more individuals with hypertension, and had higher levels of Hcy (*P* < 0.05).

### 3.2. Cognitive Psychological Assessment

The cognitive psychology of the two groups of patients was evaluated. The results showed that compared with the NC group, the scores of MoCA and CASI in the VCIND group were significantly reduced, and the scores of CDR and HAMD were increased considerably (*P* < 0.05), while the ADL scores showed no significant difference (*P* > 0.05) (Table 3).

### 3.3. MP-MRI Parameters and the Number of Genes Carried by APOE-*ε*4

**Figure 1:**
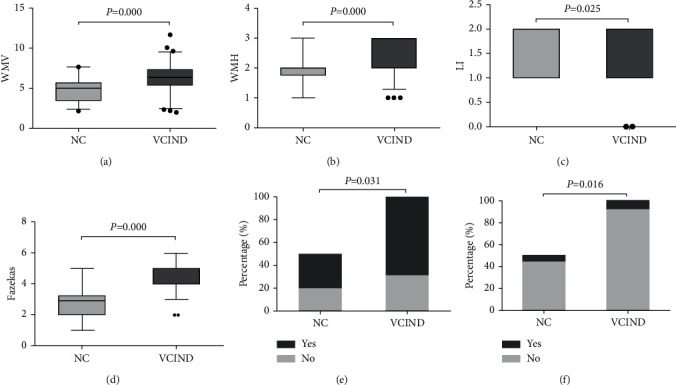
Comparison of MP-MRI parameters and APOE-*ε*4 gene carrier rate between the two groups. A, Comparison of white matter volume (WMV) between the two groups; B, comparison of white matter hyperintensity (WMH) between the two groups; C, comparison of lacunar infarction (LI) between the two groups; D, comparison of Fazekas scores between the two groups, E, the proportion of cerebral atrophy between the two groups; F, the proportion of APOE-*ε*4 gene carriage between the two groups.

Further MP-MRI examination results showed that compared with the NC group, the WMV, WMH, LI, and Fazekas scores in the VCIND group were significantly increased, and the degree of cerebral atrophy was more obvious (*P* < 0.05, Figures 1(a)–1(d)). In the VCIND group, there were 40 cases in the frontal region, 14 cases in the parieto-occipital region, 27 cases in the temporal region, and 49 cases in the basal ganglia region. In the NC group, there were 14 cases in the frontal region, 7 cases in the parieto-occipital region, 7 cases in the temporal region, and 22 cases in the basal ganglia region, indicating that leukoaraiosis was more severe in patients with VCIND (*P* < 0.05, Figure 1(e)). In addition, the results of the APOE-*ε*4 genotype test showed that compared with the NC group, the number of APOE-*ε*4 gene carriers in the VCIND group was significantly increased (*P* < 0.05, Figure 1(f)).

### 3.4. Risk Factors for VCIND Development

Risk factors for the development of VCIND were further assessed. The results of multivariate regression analysis showed that compared with the NC group, Hcy levels were higher (OR: 5.026, 95% CI: 1.198–21.089), WMV (OR: 5.026, 95% CI: 1.198–21.089), WMH (OR: 5.026, 95% CI: 1.198–21.089), and Fazekas score (OR: 5.026, 95%CI: 1.198–21.089) increased, and patients with APOE-*ε*4 gene (OR: 5.026, 95% CI: 1.198–21.089) had a significantly increased risk of VCIND (Table 4).

### 3.5. MP-MRI Parameters and APOE-*ε*4 Gene Carriage Are Related to the Occurrence of Dementia

**Figure 2:**
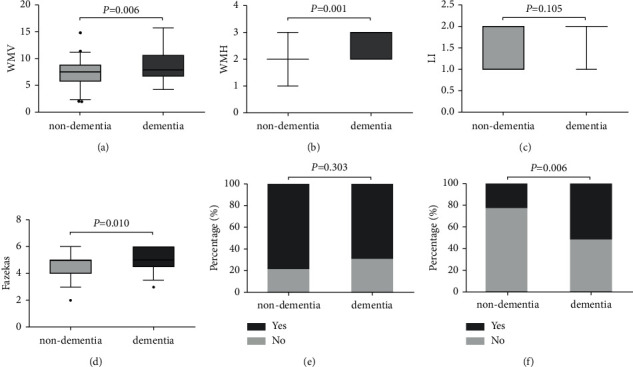
Comparison of MP-MRI parameters and APOE-*ε*4 gene carrier rate between the two groups at the 12th month of follow-up. A, Comparison of white matter volume (WMV) between the two groups at the 12th month of follow-up; B, comparison of white matter hyperintensity (WMH) between the two groups at the 12th month of follow-up; C, comparison of lacunar infarction (LI) between the two groups at the 12th month of follow-up; D, comparison of Fazekas scores between the two groups at the 12th month of follow-up, E, the proportion of cerebral atrophy between the two groups at the 12th month of follow-up; F, ratio of APOE-*ε*4 gene carriage between the two groups at the 12th month of follow-up

The association of MP-MRI parameters and APOE-*ε*4 gene with VCIND patients who developed dementia was further investigated. Through 3-month, 6-month, and 12-month follow-up, it was found that the number of patients who developed dementia gradually increased over time. The 12-month follow-up results showed that compared with the nondementia group, the WMV, WMH, and Fazekas scores in the dementia group were significantly increased; the difference between LI and cerebral atrophy was not statistically significant (*P* < 0.05, Figures 2(a)–2(d)). In the dementia group, in the leukoaraiosis sites, there were 9 cases in the frontal region, 3 cases in the parieto-occipital region, 6 cases in the temporal region, and 11 cases in the basal ganglia region. In the nondementia group, there were 31 cases in the frontal region, 11 cases in the parieto-occipital region, 21 cases in the temporal region, and 38 cases in the basal ganglia region (*P* < 0.05, Figure 2(e)). Compared with the nondementia group, the number of APOE-*ε*4 gene carriers in the dementia group was significantly increased (*P* < 0.05, Figure 2(s)). In addition, single-factor and multivariate logistic analysis of risk factors related to dementia survival rates also showed that WMV, WMH, Fazekas scores increased, and APOE-*ε*4 gene carriers were at higher risk of developing dementia (*P* < 0.05, Table 5).

**Figure 3:**
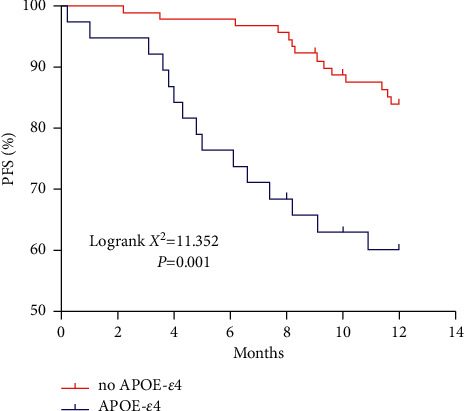
Kaplan–Meier analysis of the correlation between APOE-*ε*4 gene and survival of VCIND patients.

The relationship between the APOE-*ε*4 gene and the prognosis of patients with VCIND was further investigated. The results of Kaplan–Meier survival analysis showed that patients with the APOE-*ε*4 gene had significant progression-free survival (PFS). These results suggest that the APOE-*ε*4 gene is closely associated with VCIND disease progression (Figure 3).

## 4. Discussion

According to the Guiding Standards for the Diagnosis and Treatment of Vascular Cognitive Impairment in China (2016), VCIND is a classification of VCI in terms of disease severity. It refers to the cognitive impairment that occurs under the action of long-term vascular factors and has normal daily basic abilities. The complex instrumental daily ability is slightly impaired and does not meet the diagnostic criteria for dementia [12]. Some studies have shown that vascular risk factors directly contribute to the onset of VCIND [13]. Among them, hypertension [14] and diabetes [15] are the two most important vascular risk factors, and age is one of the most important uncontrollable factors for stroke and dementia. Hcy is an independent risk factor for the onset of cardiovascular diseases such as atherosclerosis [16]. The increase in serum Hcy level indicates the increase in the prevalence and progression of lacunar infarction. In this study, we found that compared with the NC group, patients in the VCIND group had shorter years of education, more individuals with hypertension, and higher levels of Hcy. This is consistent with the results of previous studies, namely that the two vascular risk factors, hypertension, and Hcy, promote VCIND. The MoCA score is mainly used to assess whether patients have vascular cognitive impairment after ischemic stroke. The results of Yuanbo Wu et al. [17] showed that the optimal cutoff value for MoCA to identify VCIND is 22–23; MoCA with a sensitivity of 65.26% and a specificity of 78.73%. The elementary school, middle school, and junior college groups' sensitivity was 97.06%, 56.10%, and 40%, and the specificities were 47.22%, 87.80%, and 100%, respectively. The results of this study further support previous reports in the literature that the MoCA score of VCIND patients was significantly lower than that of the NC group (15.89 ± 3.09), suggesting that educational background may have an impact on the MoCA score. In addition, the CASI scores of VCIND patients were also lower, and the CDR and HAMD scores were increased.

With the rapid development of new neuroimaging technologies, MRI has now become an important method for VCI diagnosis and research and is widely used to assess vascular changes. In this study, comparing the MRI lesions of the study subjects, it was found that the WMV, WMH, and Fazekas scores of the VCIND group were significantly higher than those of the NC group. At the same time, logistic regression analysis results showed that patients with high WMV, WMH, and Fazekas scores had a higher probability of VCIND occurrence. The follow-up results showed that compared with the nondementia group, the WMV, WMH, and Fazekas scores were significantly higher in the dementia group. These results indicate that extensive damage to white matter lesions can lead to VCIND.

As an allele of ApoE, ApoE-*ε*4 is not only the biggest genetic risk factor for AD but also related to a variety of neurological diseases. Davidson et al. [18] showed that carrying the APOE-*ε*4 allele was associated with an increased risk of vascular dementia. Treves et al. [19] showed that the APOE-*ε*4 allele frequency was highest in AD patients, followed by VCIND patients, and then the control group. Hsiung et al. [20] showed that VCIND patients and APOE-*ε*4 allele carriers are more common in advanced disease patients. This study found that compared with the NC group, the number of APOE-*ε*4 gene carriers in the VCIND group was significantly higher. Logistic regression analysis also confirmed that APOE-*ε*4 gene carriers are more likely to develop VCIND. In the process of follow-up of VCIND patients, it was found that APOE-*ε*4 gene carriers are at higher risk of developing dementia, and the progression-free survival of patients with the APOE-*ε*4 gene was significantly lower than that of noncarriers. These results confirm that APOE-*ε*4 gene carriers are closely related to the progression of VCIND disease.

However, this study has some limitations: the number of cases included is small, which may affect the accuracy of the results; APOE-*ε*4 gene may be related to region and nationality, but these factors were not considered in this study. Therefore, the number of cases and study area will be expanded in the subsequent study.

## 5. Conclusion

In summary, MP-MRI parameters and APOE-*ε*4 alleles are closely related to the occurrence and progression of VCIND, suggesting that MP-MRI and APOE-*ε*4 genotypes can be used as a diagnostic method for VCIND and provide a new research direction for the prevention and treatment of VCIND and its related diseases in clinical practice.[21–25].

## Figures and Tables

**Table 1:** Primer sequence.

Primer	Sequence
APOE-*ε*4	F：5′-TAAGCTTGGCACGGCTGTCCAAGGA-3′
	R：5′-ACAGAATTCGCCCCGGCCTGGTACAC-3′

**Table 2:** Baseline data of patients.

Features	NC group (*n* = 50)	VCIND group (*n* = 130)	*X* ^2^ */t/z*	*P*
Gender (%)			0.771	0.380
Male	26 (52.0)	77 (59.2)		
Female	24 (48.0)	53 (40.8)		
Age (years)	67.36 ± 10.95	68.65 ± 11.1	0.703	0.483
BMI (kg/m^2^）	24.20 ± 3.98	24.52 ± 4.00	0.486	0.627
Years of education	6.14 (5–7)	5.52 (5–6)	2.604	0.009
Stroke onset time (h)	8 (7–9)	8 (7–9)	0.895	0.371
Hypertension (%)			6.304	0.012
Without	25 (50.0)	39 (30.0)		
With	25 (50.0)	91 (70.0)		
Diabetes (%)			2.465	0.116
Without	37 (74.0)	80 (61.5)		
With	13 (26.0)	50 (38.5)		
Coronary heart disease (%)			0.051	0.821
Without	40 (80.0)	102 (78.5)		
With	10 (20.0)	28 (21.5)		
Smoking history (%)			0.548	0.459
Without	41 (82.0)	100 (76.9)		
With	9 (18.0)	30 (23.1)		
Drinking history (%)			0.565	0.452
Without	36 (72.0)	86 (66.2)		
With	14 (28.0)	44 (33.8)		
Fasting blood glucose	5.53 ± 0.99	5.58 ± 0.97	0.312	0.755
Triglycerides (TG）	2.27 ± 0.86	2.35 ± 0.77	0.575	0.566
Cholesterol (TC）	4.85 ± 1.07	5.10 ± 0.81	1.682	0.094
High-density lipoprotein (HDL)	1.20 ± 0.26	1.15 ± 0.28	1.044	0.298
Low-density lipoprotein (LDL）	2.76 ± 1.09	2.89 ± 0.95	0.792	0.429
Homocysteine (hcy) (%）			14.014	≤0.001
＜13.9 mol/L	40 (80.0)	64 (49.2)		
≥13.9 mol/L	10 (20.0)	66 (50.8)		

Values are mean ± SD or *n* (%) or median (P25, P75). NC, normal control; VCIND, vascular cognitive impairment no dementia.

**Table 3:** Comparison of cognitive psychology assessment between the two groups of patients.

Features	NC group (*n* = 50)	VCIND group (*n* = 130)	*t/z*	*P*
Clinical Dementia Rating (CDR）	8 (7–9)	12 (11–14)	8.993	≤0.001
Activity of Daily Living Scale (ADL)	13 (10–16)	13.5 (12–16)	1.924	0.054
Montreal Cognitive Assessment (MoCA)	19.74 ± 2.93	15.89 ± 3.09	7.589	≤0.001
Elderly Cognitive Abilities Screening Instrument (CASI)	24 (23–25)	17 (15–19)	9.783	≤0.001
Hamilton Rating Scale For Depression (HAMD)	9 (6–10)	16 (12–18)	8.860	≤0.001

Values are mean ± SD or median (P25, P75). NC, normal control; VCIND, vascular cognitive impairment no dementia.

**Table 4:** Single-factor and multivariate logistic regression analysis of VCIND-related risk factors.

Features	Single factor		Multivariate	
	OR (95% CI)	*P*-value	OR (95% CI)	*P*-value
Age	1.011 (0.981–1.041)	0.481		
Gender	0.746 (0.387–1.437)	0.380		
BMI	1.021 (0.940–1.108)	0.625		
Years of education	0.645 (0.498–0.834)	0.001	0.696 (0.456–1.061)	0.092
Stroke onset time	0.931 (0.778–1.114)	0.434		
Hypertension	2.333 (1.195–4.557)	0.013	2.066 (0.592–7.204)	0.255
Diabetes	1.779 (0.862–3.669)	0.119		
Coronary heart disease	1.098 (0.489–2.467)	0.821		
Smoking history	1.367 (0.597–3.131)	0.460		
Drinking history	1.316 (0.643–2.693)	0.453		
Fasting blood glucose	1.055 (0.754–1.478)	0.754		
Triglycerides (TG)	1.130 (0.747–1.709)	0.560		
Cholesterol (TC)	1.369 (0.945–1.984)	0.096		
High-density lipoprotein (HDL)	0.524 (0.156–1.765)	0.297		
Low-density lipoprotein (LDL)	1.145 (0.820–1.600)	0.427		
Homocysteine (hcy)	4.125 (1.903–8.941)	0.000	5.026 (1.198–21.089)	0.027
White matter volume (WMV)	1.843 (1.438–2.361)	0.000	1.549 (1.072–2.240)	0.020
White matter hyperintensity (WMH)	8.947 (3.448–23.219)	0.000	5.199 (1.261–21.44)	0.023
Lacunar infarction (LI)	2.013 (1.118–3.623)	0.020	1.823 (0.583–5.705)	0.302
Fazekas score	5.473 (3.376–8.874)	0.000	4.631 (2.615–8.203)	≤0.001
Leukoaraiosis region				
Frontal region (reference group)	1.000			
Parieto-occipital region	0.700 (0.235–2.087)	0.522		
Temporal region	1.350 (0.482–3.783)	0.568		
Basal ganglia region	0.780 (0.354–1.717)	0.536		
Cerebral atrophy	2.129 (1.063–4.265)	0.033	2.882 (0.780–10.647)	0.112
*ε*4 gene carrier	3.029 (1.192–7.700)	0.020	6.150 (1.164–32.482)	0.032

**Table 5:** Single-factor and multivariate logistic analysis of risk factors related to dementia.

Features	Single factor		Multivariate	
	HR (95% CI)	*P*-value	HR (95% CI)	*P*-value
Age	0.974 (0.943–1.007)	0.118		
Gender	1.638 (0.790–3.396)	0.185		
BMI	1.040 (0.949–1.140)	0.397		
Years of education	0.913 (0.691–1.206)	0.521		
Stroke onset time	0.943 (0.781–1.140)	0.545		
Hypertension	0.699 (0.330–1.480)	0.349		
Diabetes	0.979 (0.462–2.073)	0.956		
Coronary heart disease	0.399 (0.121–1.318)	0.132		
Smoking history	1.336 (0.592–3.017)	0.486		
Drinking history	1.941 (0.936–4.024)	0.075		
Fasting blood glucose	0.916 (0.618–1.358)	0.662		
Triglycerides (TG)	1.503 (0.903–2.500)	0.117		
Cholesterol (TC)	1.107 (0.708–1.731)	0.657		
High-density lipoprotein (HDL)	1.714 (0.446–6.590)	0.433		
Low-density lipoprotein (LDL)	0.691 (0.465–1.027)	0.067		
Homocysteine (hcy)	0.862 (0.416–1.786)	0.690		
White matter volume (WMV)	1.329 (1.129–1.564)	0.001	1.280 (1.073–1.528)	0.006
White matter hyperintensity (WMH)	2.742 (1.413–5.322)	0.003	3.044 (1.512–6.129)	0.002
Lacunar infarction (LI)	1.946 (0.804–4.709)	0.140		
Fazekas score	1.841 (1.150–2.947)	0.011	1.984 (1.231–3.198)	0.005
Leukoaraiosis region				
Frontal region (reference group)	1.000			
Parieto-occipital region	0.953 (0.258–3.520)	0.942		
Temporal region	1.052 (0.374–2.957)	0.923		
Basal ganglia region	0.971 (0.402–2.343)	0.948		
Cerebral atrophy	0.633 (0.288–1.391)	0.255		
*ε*4 gene carrier	3.267 (1.575–6.776)	0.001	2.442 (1.115–5.35)	0.026

Carriage of the APOE-*ε*4 gene is associated with survival in patients with VCIND.

## Data Availability

The data used to support the findings of this study are available from the corresponding author upon request.
